# French Report on Vaccination

**Published:** 1845-09

**Authors:** 


*French Report on Vaccination.*—This report was read by M. Serres at the seances of the Royal Academy of Sciences, on the 21st of February and 3rd of March. The following are the general conclusions with which it terminates.

1. The preservative power of Vaccination is absolute in a large majority of individuals, and temporary only in a small number. Even in the latter case, it is almost absolute up to the period of adolescence.

2. Small-pox rarely attacks vaccinated persons before the 10th or 12th year of their age. From this period to the 30th or 35th year of life, the liability to variolous infection is greatest.

3. Besides its preservative virtue, Vaccination introduces into the organisation a property or power which attenuates (so to speak) the symptoms of small-pox, shortens their duration, and very considerably diminishes their severity and danger.

4. Cow-pox lymph, taken directly from the animal, gives to the local phenomena of Vaccination a more decided intensity, and its effects are more certain than when old virus has been used. It would seem, however, that, in the course of a few weeks after its transmission to the human system, this local intensity no longer exists.

5. The preservative power of cow-pox does not appear to be intimately connected with the intensity of the symptoms induced by Vaccination: nevertheless, in order that the properties of the lymph be duly preserved, it will be prudent to regenerate it as frequently as possible.

6. Among the means that have been proposed to effect this regeneration, the only one deserving of confidence is that of deriving the vaccine lymph occasionally from its parent source.

7. Re-vaccination is the only sure test that we have to enable us to distinguish such vaccinated persons as are decidedly proof against the infection of small-pox, from those that are only partially or imperfectly so.

8. The result of re-vaccination does not afford a certain proof that those vaccinated persons, in whom it takes effect, were destined to contract small-pox; but only a high probability, that it is especially among such individuals that the disease is likely to occur.

9. As a general rule, re-vaccination should be practised at, and from, the 14th year of life. If small-pox, however, be epidemic, it will be prudent to anticipate this age, and re-vaccinate from the 8th or 9th year.

Among the prefatory observations of the report, allusion is made to the circumstance that Vaccination will sometimes *take* most perfectly in persons who have had small-pox. M. Moreau, the distinguished accoucheur of Paris, assures us that he has vaccinated himself with effect three different times, although he had the small-pox in his youth.

An official document, published by the Government of Wurtemburg, and wherein it is set forth that, out of 1677 cases of small-pox which occurred from the year 1831 to 1836, no fewer than 1055 were in vaccinated persons, contributed very materially to encourage the performance of re-vaccination in most parts of Germany.

The reports, too, of various epidemics of small-pox of late years in this country (France) clearly shew that the proportion of vaccinated persons, who have become affected with the disease, is more than one-third of the entire number attacked. The importance, or rather the necessity, of re-vaccination is therefore strikingly apparent. We have good reason for believing not only that multitudes have been preserved from variolous contagion by having recourse to this measure, but also that the disease has thus been actually arrested in its progress by, as it were, a barrier which it could not overleap.

The effects of re-vaccination in the Prussian army, since the year 1833, have almost completely extirpated small-pox from its ranks. In the Kingdom of Wurtemburg also, it has been found that out of 14,384 soldiers and 19,864 civilians, who were re-vaccinated, only one case of Varioloid has occurred among the former, and only three among the latter, during a period of five years.

Since the year 1830, when the practice of re-vaccination became general, no epidemic of small-pox has been experienced in that kingdom. The good effects of this practice have been not less striking in some of the Italian states.—*Med. Chir. Rev. from Comptes Rendus.*

